# Brain microvascular endothelial cell dysfunction during *Plasmodium* infection does not result from astrocyte-derived wingless-related integration site (Wnt)7a deficiency

**DOI:** 10.64898/2026.10.18.760006

**Published:** 2026-10-23

**Authors:** Marshall Roedel, Nia Brooks, Jared N. Andersen, Douglas H. Cornwall, Rohan Doddavaram, Kennedy Chastang, Leah Rubner, Tao Liu, Tracey J. Lamb

## Abstract

Breakdown of the blood brain barrier is a key event leading to vasogenic edema that underpins pediatric cerebral malaria. Brain microvascular endothelial cells (BMECs) downregulate junction proteins in response to *Plasmodium* -infected red blood cells (iRBCs). Very little is known about how glial cell activation in *Plasmodium* infection dysregulates astrocyte-derived steady state signals that control BMEC junction expression. Astrocytes from mice infected with *Plasmodium berghei* ANKA ( *Pb* A) exhibit transcriptional changes dominated by interferon signaling pathways. Downregulation of wingless-related integration site (Wnt)7a transcription in astrocytes correlated with transcriptional changes in BMECs indicative of reduced *β* -catenin activity. Incubation of the human astrocyte-like glioma cell-line CCF-STTG1 with interferon-γ (IFN-γ) decreased the general production of Wnt7a in response to *P. falciparum* iRBCs. However astrocyte conditioned medium incubated with IFN-γ strengthened the barrier function *in vitro* when added onto monolayers the human cerebral microvascular endothelial cell line D3 (hCMEC/D3). This data suggests that the response of astrocytes during *Plasmodium* infection may be protective but unable to overcome dysfunctional responses of BMECs to *Plasmodium* iRBCs. To test this hypothesis we overexpressed Wnt7a in the brain tissue of mice using an AAV construct but this did not impart increased survival to the neurological effects of *Pb* A infection. Therefore astrocyte responses during *Plasmodium* infection may not underlie the downregulation of junction proteins on BMECs leading to edema in cerebral malaria.

## Full Text Availability

The license terms selected by the author(s) for this preprint version do not permit archiving in PMC. The full text is available from the preprint server.

